# Is CRT pro-arrhythmic? A comparative analysis of the occurrence of ventricular arrhythmias between patients implanted with CRTs and ICDs

**DOI:** 10.3389/fphys.2014.00334

**Published:** 2014-09-17

**Authors:** A. B. Gopalamurugan, G. Ganesha Babu, Dominic P. Rogers, Adam L. Simpson, Syed Y. Ahsan, Pier D. Lambiase, Anthony W. Chow, Martin D. Lowe, Edward Rowland, Oliver R. Segal

**Affiliations:** Department of Cardiac Electrophysiology, The Heart Hospital, Institute of Cardiovascular Sciences, University College LondonUK

**Keywords:** cardiac resynchronization therapy, ICD, heart failure, ventricular arrhythmia

## Abstract

**Aim and Hypothesis:** Despite the proven symptomatic and mortality benefit of cardiac resynchronization therapy (CRT), there is anecdotal evidence it may be pro-arrhythmic in some patients. We aimed to identify if there were significant differences in the incidence of ventricular arrhythmias (VAs) in patients undergoing CRT-D and implantable cardioverter-defibrillators (ICD) implantation for primary prevention indication. We hypothesized that CRT is unlikely to be pro-arrhythmic based on the positive mortality and morbidity data from large randomized trials.

**Methods and Results:** A retrospective analysis of device therapies for VA in a primary prevention device cohort was performed. Patients with ischemic (IHD) and non-ischemic (DCM) cardiomyopathy and ICD or CRT+ICD devices (CRT-D) implanted between 2005 and 2007 without prior history of sustained VA were included for analysis. VA episodes were identified from stored electrograms and defined as sustained (VT/VF) if therapy [anti-tachycardia pacing (ATP) or shocks] was delivered or non-sustained (NSVT) if not. Of a total of 180 patients, 117 (68% male) were in the CRT-D group, 42% IHD, ejection fraction (EF) 24.5 ± 8.2% and mean follow-up 23.9 ± 9.8 months. 63 patients (84% male) were in the ICD group, 60% IHD, EF 27.7 ± 7.2% and mean follow-up 24.6 ± 10.8 months. Overall, there was no significant difference in the incidence of VA (35.0 vs. 38.1%, *p* = 0.74), sustained VT (21.3 vs. 28.5%, *p* = 0.36) or NSVT (12.8 vs. 9.5%, *p* = 0.63) and no significant difference in type of therapy received for VT/VF: ATP (68 vs. 66.6%, *p* = 0.73) and shocks (32 vs. 33.3%, *p* = 0.71) between the CRT-D and ICD groups, respectively.

**Conclusion:** In patients with cardiomyopathy receiving CRT-D and ICDs for primary prophylaxis, there was no significant difference in the incidence of VA. From this single center retrospective analysis, there is no evidence to support cardiac resynchronization causing pro-arrhythmia.

## Introduction

**Figure 1:**
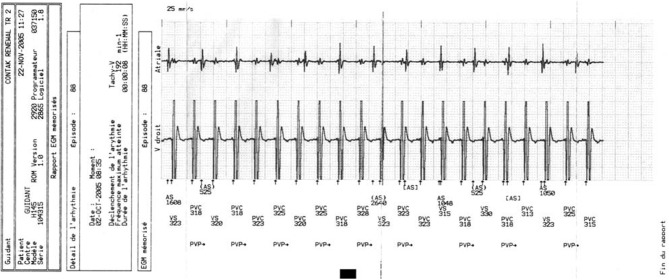
**A device interrogation printout showing ventricular tachycardia**.

Cardiac resynchronization therapy (CRT) is an established treatment for chronic heart failure with proven mortality and morbidity benefits (Abraham et al., 2002; Cohen and Klein, 2002; John Sutton et al., 2003). In patients with heart failure and left bundle branch block on surface ECG, CRT reduces symptoms, hospitalization and sudden death (Cazeau et al., 2001; Abraham et al., 2002; Higgins et al., 2003). Patients with congestive heart failure are at increased risk of developing both monomorphic ventricular tachycardia (VT) (Figure 1) and ventricular fibrillation (VF) (Stevenson and Stevenson, 2001). Re-entry within the left ventricle (LV) is responsible for the majority of VT circuits (Stevenson and Delacretaz, 2000). In selected patients, implantable cardioverter defibrillator (ICD) implantation, singly or in combination with CRT (CRT-D), is indicated for primary or secondary prevention purposes, with proven mortality benefit and reduced risk of arrhythmic death for both device type (Walker et al., 2000; Zagrodzky et al., 2001; Martinelli et al., 2002; Higgins et al., 2003; Bristow et al., 2004; Cleland et al., 2005; Voigt et al., 2006). Based on the available data we hypothesized that CRT is unlikely to be pro-arrhythmic. However, there have also been numerous reports of pro-arrhythmia in patients treated with CRT, questioning its safety (Medina-Ravell et al., 2003; Di Cori et al., 2005; Fish et al., 2005; Shukla et al., 2005; Germano et al., 2006; Spragg and Kass, 2006). Various mechanisms for this phenomenon have been postulated, including reversal of the normal transmural sequence of activation, QT prolongation and increasing transmural dispersion of repolarization but much of these data are derived from *in-vitro* studies (Fish et al., 2005) and there has been a lack of clinical studies designed to investigate this problem in more detail. Given the conflicting evidence for the pro-arrhythmic effects of CRT and the lack of clinical data, the aim of the present study was to investigate and compare the incidence of ventricular arrhythmias (VAs) in patients with either CRT/CRT-D or ICD devices indicated for primary prevention implanted in our center.

## Methods

This study is a single center retrospective analysis of patients with ICD and CRT/CRT-D devices implanted between January 2005 and December 2007. All patients had ischemic or non-ischemic cardiomyopathy (idiopathic dilated cardiomyopathy) and poor left ventricular function. Patients with hypertrophic cardiomyopathy, infiltrative cardiomyopathy, ion-channelopathies, and congenital heart disease were excluded from the study. A total of 840 patients' device implantation records and indications were analyzed. Patients with devices implanted for secondary prevention of VA and those with a separate bradycardia pacing indication were excluded. Patients with normal left ventricular ejection fraction (EF) were excluded from the study. Demographic and clinical data, including renal function, were identified from patient records. Baseline renal function was classified using the estimated glomerular filtration rate (eGFR) by Modification of Diet in Renal Disease (MDRD) formula (Levey et al., 1999). Patients with eGFR < 60 mls/min/1.73 m^2^ were classified as having chronic kidney disease (CKD) stage 3 or worse (National Kidney Foundation, 2002).

The cohort was divided into an ICD group and a CRT-D group. After exclusions, a total of 180 patients' device records were included. Details of episodes of VAs were obtained from patient notes, device clinic records and analysis of stored electrograms, downloaded device data from the previous 2.5 years, if available, in all patients.

### Definitions

Device therapy was classified into either anti-tachycardia pacing (ATP) or shocks. Patients who received a combination of ATP and shocks for a single arrhythmia episode were classified in the “shock” category. VAs were defined as a VT or VF episode meeting device detection criteria, which then led to therapy delivery. Non-sustained ventricular tachycardia (NSVT) was defined as a VA episode detected by the device without therapy delivery due to spontaneous termination. Therapies delivered due to atrial fibrillation (AF) or supraventricular tachycardia were deemed inappropriate and were not included for analysis.

Patients had devices from the following manufacturers: Medtronic, Boston Scientific, St Jude and Biotronik. Criteria for detection of VAs were programmed to nominal settings at implant and were altered only at their physician's discretion. This also applied to therapy programming.

## Statistics

Categorical variables are presented as percentages and continuous variables as mean ± SD and/or median (interquartile range) where appropriate. Categorical variables were compared by the *X*^2^-test or Fisher's exact test where applicable. Kaplan-Meier analysis was performed for survival probability and log rank test was used for time to event comparison. A *P*-value < 0.05 was considered significant. Statistical comparisons were done using statistical software SPSS 15 (Manufacturer name, city, USA).

## Results

### Patient characteristics

Between 2005 and 2007, 63 patients in the ICD group and 117 patients in the CRT-D group were identified after exclusion criteria. Mean follow-up was 24.6 ± 10.8 months in the ICD group and 23.9 ± 9.8 in the CRT-D group. Baseline patient characteristics are shown in Table 1.

**Table 1:** **Patient characteristics of both groups (ACE, Angiotension Converting Enzyme; ARB, Angiotensin Receptor Blockers; CKD, Chronic Kidney Disease; NYHA, New York Heart Association)**.

**Patient characteristics**	**CRT-D group (*n* = 117)**	**ICD group (*n* = 63)**	**Significance**
Age	66 ± 11	65.3 ± 13	>0.05
Gender			
Male	80 (68%)	53 (84%)	0.02
Female	37 (32%)	10 (16%)	
Etiology			
Ischemic cardiomyopathy	49 (42%)	38 (60%)	0.03
Non-ischemic ardiomyopathy	68 (58%)	25 (40%)	>0.05
Ejection fraction (%)	24.5 ± 8.2	27.7 ± 7.2	>0.05
Follow-up (months)	23.9 ± 9.8	24.6 ± 10.8	>0.05
NYHA class (median)	III	II	0.0003
ACE inhibitors or ARBs	97%	92%	>0.05
Amiodarone	14%	25%	>0.05
Beta blockers	86%	69%	0.026
CKD stage 3 or above	46%	36%	>0.05

Patients in the CRT-D group had standard indications, including LVEF ≤35%, NYHA class III-IV symptoms and QRS duration >150 ms or QRS >120 ms and echocardiographic evidence of dyssynchrony. All patients were on the maximum tolerated heart failure therapy. Of the 117 patients in the CRT-D group, 80 (68%) were male, mean age 66 ± 11 years and of the 63 patients in the ICD group 53 (84%) were male, with a mean age of 65.3 ± 13 years. The etiology of the underlying LV dysfunction in the CRT-D group was ischemic in 49 (42%) patients and non-ischemic cardiomyopathy in 68 (58%) patients. In the ICD group, 38 (60%) patients had ischemic cardiomyopathy. Mean LV EF was 24.5 ± 8.2% in the CRT-D group and 27.7 ± 7.2% in the ICD group. Mean follow-up was 23.9 ± 9.8 months and 24.6 ± 10.8 months in the CRT-D and ICD groups, respectively. The median NYHA class of patients in the CRT-D group was 3 and was class 2 in the ICD group. 97% of patients were established on an ACE inhibitor or Angiotensin receptor blocker in the CRT-D group and 92% in the ICD group. 14% in the CRT-D group were on Amiodarone and 25% in the ICD group. In the CRT-D group 86% were on a beta-blocker and 69% in the ICD group. Analysis of patients' renal function revealed 46% of patients had CKD stage 3 or above in the CRT-D group and 36% of patients in the ICD group. Both groups were well matched for age, etiology, EF, follow up duration, ACE inhibitor/ARB use and the presence of CKD Stage 3 or more (all *p* = NS) (see Table 1). Disparity was seen between the groups for NYHA class (worse in CRT-D group), gender (more females CRT-D group), number of patients with ischemic cardiomyopathy (less ischemic cardiomyopathy in CRT-D group) and beta-blocker usage (higher use in CRT-D group).

### Incidence of ventricular arrhythmias

**Figure 2:**
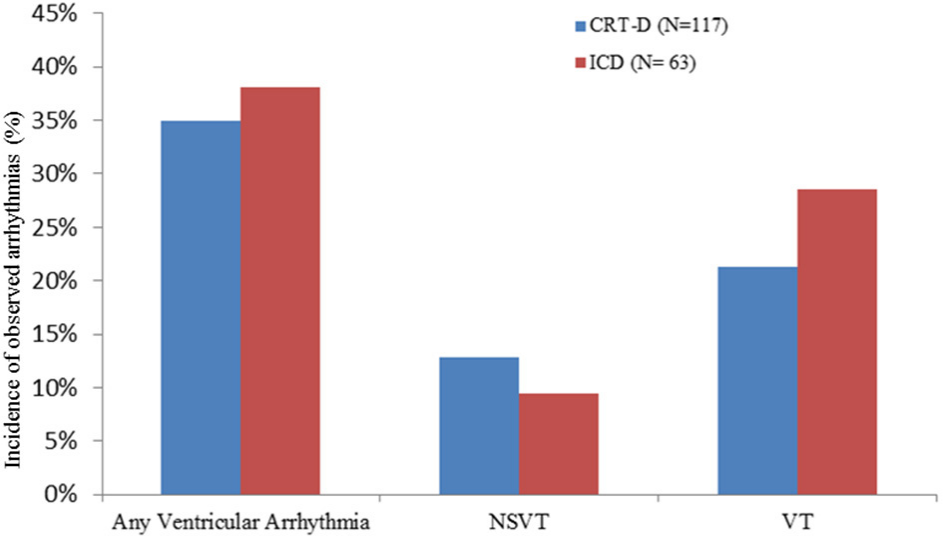
**Incidence of tachy-arrhythmias in the CRT-D and ICD groups**.

In the CRT-D group, 35% of patients (41/117) exhibited VAs, and of these, 25 (60.9%) were VT and 16 (39.02%) were NSVT. Among those patients with VT, 17 (68%) were treated with ATP and 6 (24%) with shocks, 2 (8%) were treated with ATP followed by a shock. In the ICD group 24/63 (38.1%) patients had VAs, of which18 (75%) were VT and 6 (25%) were NSVT. Among the patients who had VT, 12 (66.6%) were treated with ATP and 6 (33.3%) with shocks. There was no significant difference in the incidence of VAs between the CRT-D and ICD groups (*p* = 0.74). There were no significant differences in the incidence of VT (*p* = 0.36) and NSVT (*p* = 0.63), nor was there a significant difference in the number of patients who received ATP or shocks (*p* = 0.714) in either groups (Figure 2).

**Figure 3:**
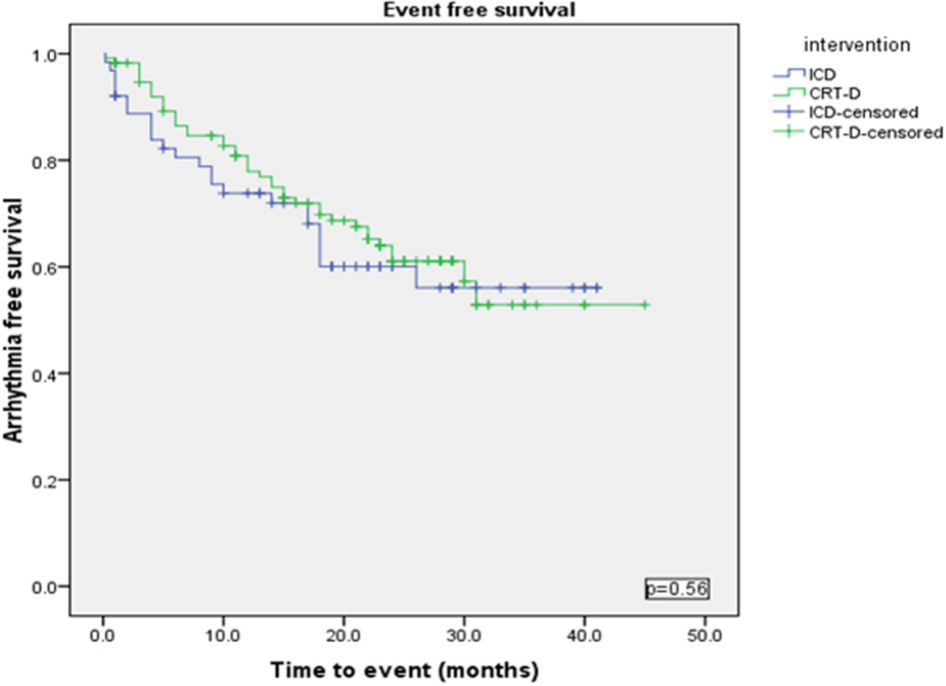
**Kaplan Meyer survival analysis of the occurrence of ventricular tachyarrhythmia between ICD and CRT-D group**.

**Figure 4:**
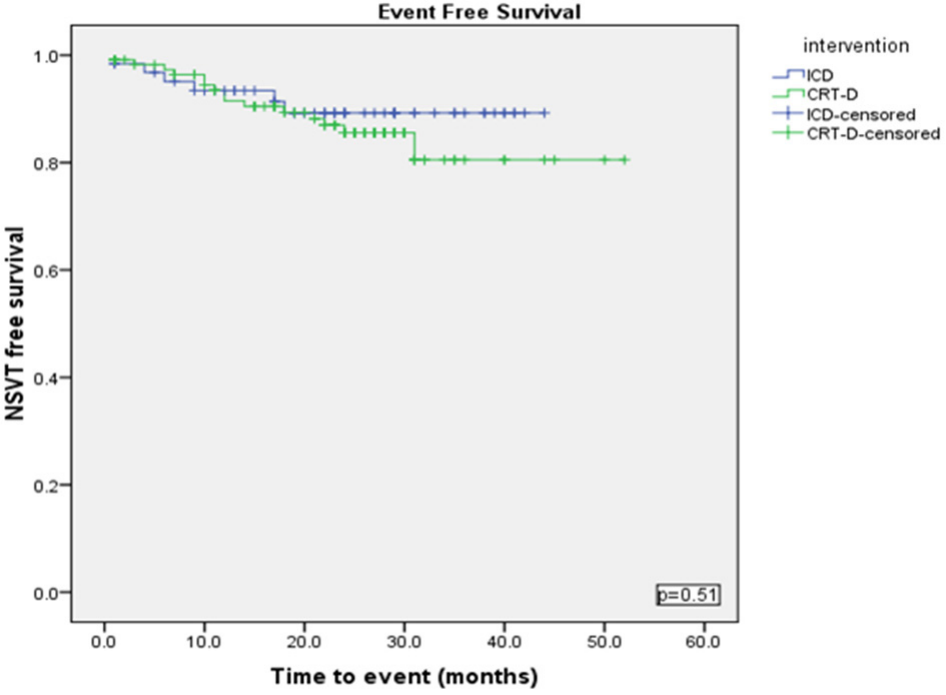
**Kaplan Meyer survival analysis of the occurrence of NSVT between ICD and CRT-D groups**.

**Figure 5:**
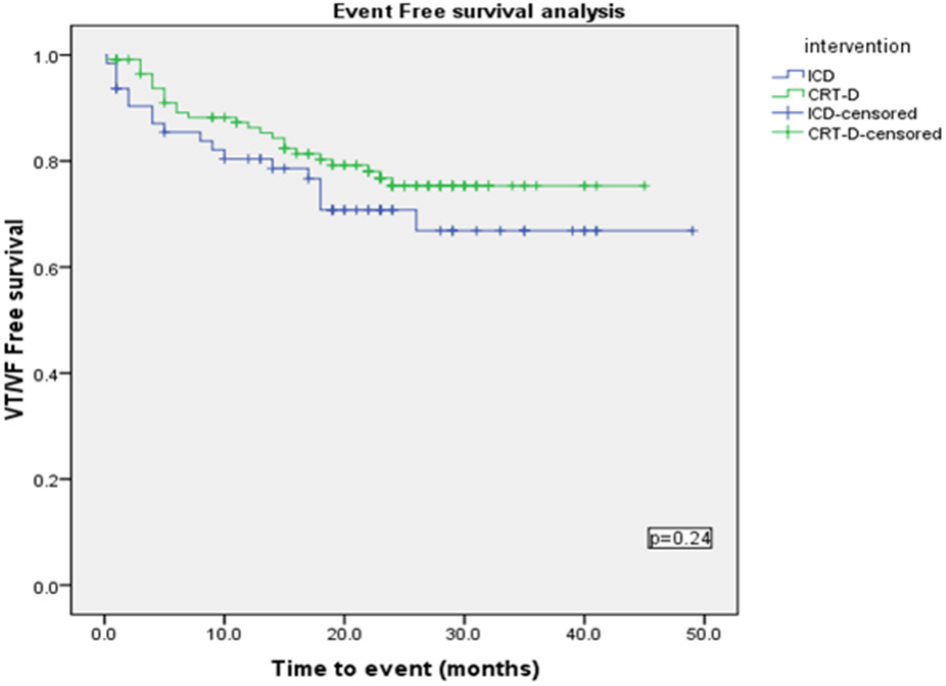
**Kaplan Meyer survival analysis of the occurrence of VT/VF between ICD and CRT-D groups**.

Using a Kaplan-Meier survival analysis, no significant difference in event free survival probability between the two groups were identified for freedom from VA, VT/VF or NSVT (Figures 3–5).

A Cox regression analysis was performed using Backward stepwise Ward rule for the occurrence of VT/VF with covariates—Age, Sex, type of intervention (ICD or CRT-D), Use of Amiodarone, Beta blocker, ACE inhibitor, NYHA Class and mean EF at the time of device implant and CKD. This showed that Male Sex (*p* = 0.001), presence of CKD (eGFR < 60) (*p* = 0.01) and Age (*p* = 0.012) to be significant predictors for Time to VT/VF.

### Sub-group analysis based on etiology

A further sub-group analysis was performed according to etiology of left ventricular dysfunction.

#### Ischemic cardiomyopathy sub-group

**Figure 6:**
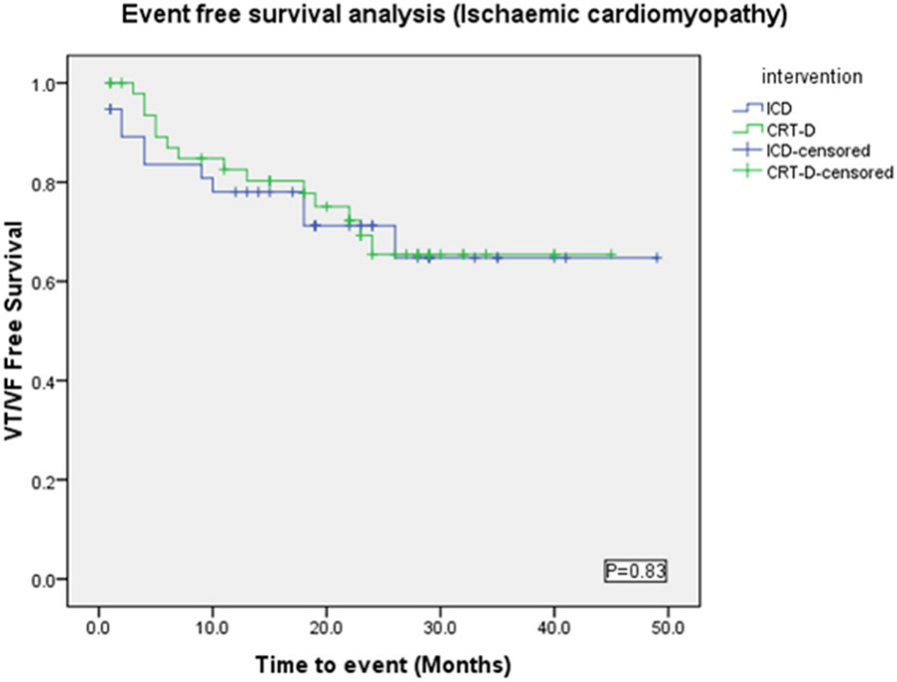
**Kaplan Meyer survival analysis of the occurrence of VT/VF between ICD and CRT-D groups in the ischemic cardiomyopathy patient cohort**.

Baseline characteristics were similar between both groups (Table 2), except NYHA class (*p* = 0.0003) and numbers of patients on Amiodarone (*p* = 0.02) or Beta-blockers (*p* = 0.03). VAs occurred in 21/49 (42%) in the CRT-D group vs. 16/38 (42%) in the ICD group (*p* = 0.94). VT/VF occurred in 14/49 (28.5%) patients in the CRT-D group vs. 11/38 (28.9%) in the ICD group (*p* = 0.96) (Figure 6).

**Table 2:** **Ischemic cardiomyopathy—Patient characteristics**.

**Patient characteristics**	**CRT-D group (*n* = 49)**	**ICD group (*n* = 38)**	**Significance**
Age	67.5 ± 9	71.4 ± 9	0.60
Gender			
Male	42 (85%)	31 (81%)	0.77
Ejection fraction (%)	26.08 ± 7.7	27.0 ± 6.3	0.56
Follow-up (months)	24.24 ± 10.4	24.79 ± 11.1	0.81
NYHA class (median)	III	II	0.0003
ACE inhibitors or ARBs	96% (36)	95% (30)	0.96
Amiodarone	13% (5)	37% (12)	0.02
Beta blockers	60% (23)	84% (27)	0.03
CKD stage 3 or more	59% (29)	46% (17)	0.27

#### Non-ischemic cardiomyopathy sub-group

**Figure 7:**
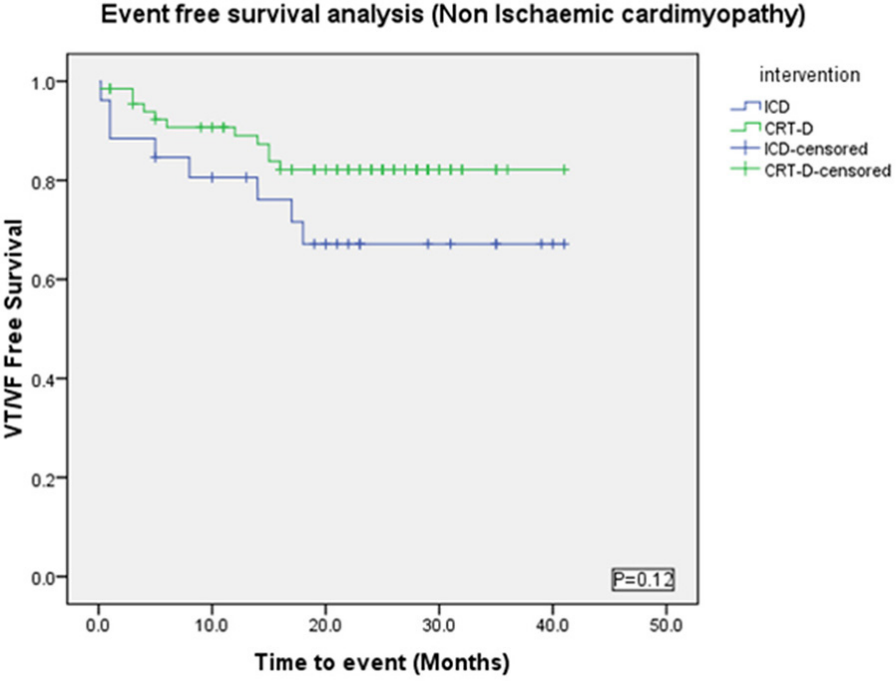
**Kaplan Meyer survival analysis of the occurrence of VT/VF between ICD and CRT-D groups in the Non-Ischemic Cardiomyopathy patient cohort**.

There were more males in the ICD group (88%) than the CRT-D group (55%) (*p* = 0.002) (Table 3). VAs occurred in 20/68 (29%) patients in the CRT-D group vs. 9/26 (35%) in the ICD group (*p* = 0.62). Sustained VA (VT/VF) occurred in 11/68 (16%) patients in the CRT-D group vs. 8/26 (31%) in the ICD group (*p* = 0.15), implying a trend toward a higher incidence of sustained VA in patients with ICDs although statistically not significant (Figure 7).

**Table 3:** **Non-ischemic cardiomyopathy—Patient characteristics**.

**Patient characteristics**	**CRT-D group (*n* = 68)**	**ICD group (*n* = 26)**	**Significance**
Age	63.6 ± 13	61.3 ± 14	0.46
Gender			
Male	55% (38)	88% (23)	0.002
Ejection fraction (%)	23.4 ± 8.5	28.6 ± 9.0	0.011
Follow-up (months)	23.7 ± 9.5	23.8 ± 10.7	0.9
NYHA class (median)	III	II	0.0003
ACE inhibitors or ARBs	75% (51)	80% (21)	0.18
Amiodarone	12% (8)	4% (1)	0.43
Beta blockers	75% (41)	90% (19)	0.2
CKD stage 3 or more	36% (25)	23% (6)	0.22

After stratifying into Ischemic and Non Ischemic Cardiomyopathy, a Cox regression analysis was performed using Backward stepwise Ward rule for the occurrence of VT/VF with covariates—Age, Sex, type of intervention (ICD or CRT-D), Use of Amiodarone, Beta blocker, ACE inhibitor, NYHA Class and mean EF at the time of device implant and presence of CKD.

Age (*p* = 0.08) and Male sex (*p* = 0.027) were found to be independent predictor for time to VT/VF in Ischemic Cardiomyopathy group. In the NICM group, Age (*p* = 0.01), Sex (*p* = 0.04), Presence of CKD (*p* = 0.01) and Intervention type- ICD (*p* = 0.03) were independent predictors of time to VT/VF occurrence.

## Discussion

The principal finding of the present study was the observation of no difference in burden of VA in patients implanted with ICDs or CRT-Ds for primary prevention purposes, in a reasonably well matched cohort. To our knowledge, this is the first retrospective study designed to specifically examine the difference in incidence of any VAs between these patient groups. In addition, there were no significant difference in the occurrence of sustained VA in the two groups (21.3% in CRT-D group vs. 28.5% in ICD group, *p* = 0.36). Sub-group analysis showed similar reductions in VAs in different etiologies of underlying cardiomyopathy. These results are supported by data from the COMPANION trial (Comparison of Medical Therapy, Pacing, and Defibrillation in Chronic Heart Failure study) (Bristow et al., 2004), which showed a significant reduction in all cause mortality and hospitalizations in patients receiving CRT or CRT-D devices compared to optimal medical therapy alone. The latter is important as one would assume an increase in sustained VAs would have led to ICD therapies resulting in increased hospitalizations in these patients. This is corroborated by data from the Cardiac Resynchronization in Heart Failure study (CARE-HF) of patients receiving CRT compared to medical therapy (Cleland et al., 2005), which showed a 37% reduction in all cause mortality and hospitalizations, but also significant improvement in patients' quality of life at 90 day follow-up. This too implies a reduction in ventricular tachyarrhythmia burden.

### Mechanisms by which CRT may be pro- or anti-arrhythmic

It is well known that CRT may cause reverse mechanical and electrical remodeling of the LV (Saxon et al., 2002; Auricchio and Abraham, 2004; Leclercq and Hare, 2004). At a sub-cellular level, the favorable role of CRT in re-organization of sub-cellular structure and protein distribution associated with excitation contraction (EC) coupling has been shown by Sachse et al. (2012) The role of sub-cellular structure transverse tubular system (t-system) along with spatial orientation of Ryanodine receptor clusters is an important requisite in EC coupling, which is invariably altered in dyssynchronous heart failure (Louch et al., 2010). CRT has been shown to restore structure and functional restitution of the t-system as early as 3 weeks in canine model (Sachse et al., 2012). The role of miRNA down regulation in heart failure patients and CRT and miRNA response to CRT in both responders and non-responders to CRT have been recently shown by Sardu et al. focusing on the miR-mediated modulation of cardiac angiogenesis, apoptosis, fibrosis and membrane ionic currents (Sardu et al., 2014). Possible mechanisms for reduction in VA with CRT include a decrease in local conduction delay, decreased pause dependant ectopic firing and decreased circulating catecholamine levels through hemodynamic effects (Zagrodzky et al., 2001). Over time, electrical and mechanical remodeling of the ventricles might exert anti-arrhythmic effects. In the MADIT-CRT trial, “super-responders” to CRT-D (with reductions of >25% in left ventricular end-systolic volume) had significantly lower VA compared to non-responders and ICD-only patients (Barsheshet et al., 2011). CRT is also associated with favorable reverse remodeling effects on the left atrium (LA), in addition to its well-established effects on the LV, thereby possibly reducing the risk for the development of Atrial tachycardia (AT) and AF in patients with HF (Kies et al., 2006; Fung et al., 2008). In CRT responders, significant improvement in left atrial (LA) functional, structural and anatomic remodeling have been shown in detailed echocardiographic assessments during follow-up (Donal et al., 2009). In addition, CRT responders have been shown to develop much lower incidence of AF during follow up period compared to non-responders (D'Ascia et al., 2011). In the MADIT-CRT trial, favorable reverse remodeling of the LA with CRT-D therapy was associated with a significant reduction in risk of subsequent AT (Brenyo et al., 2011). Although aforementioned studies and few other small studies mostly observational have reported decreased atrial arrhythmic incidence in CRT patients, a *post-hoc* analysis of large randomized CARE-HF study failed to show any atrial anti-arrhythmic effect of CRT (Hoppe et al., 2006). These studies have been evaluated in a recent meta-analysis by Hess et al. (2013)

The effects of CRT may also potentially be pro-arrhythmic. Pacing the LV *epicardially* results in non-physiological direction of activation which can lead to prolongation of action potential duration, potentially leading to prolongation of repolarization (Medina-Ravell et al., 2003; Fish et al., 2005). In patients with diseased ventricles with heterogeneous conduction, re-entry may be facilitated by pacing close to scar border or lines of block or may provoke ectopy leading to arrhythmia (Fish et al., 2005).

How the balance of these potential pro- and anti-arrhythmic mechanisms lies in different groups of patients, different etiologies of heart disease, and different conduction defects is unknown. Data from the present study and previous large, randomized trials (Voigt et al., 2006; Barsheshet et al., 2011) would support a net beneficial effect overall, but whether this is true for each individual patient is also undetermined. Previous reports of VT storm occurring acutely after CRT implantation demonstrate this balance can be difficult to predict (Kantharia et al., 2006; Nayak et al., 2008) and may simply reflect the lack of time for reverse remodeling to occur with CRT in some individuals. One can speculate that as this is only seen in a small fraction of patients, unusual interplay of specific characteristics may be responsible. LV structural abnormalities, local conduction delay and repolarization abnormalities, LV lead position and pacing site, circulating catecholamine levels as a result of hemodynamic status, medications and ion channel function may all be culprits. Finally, patients with indications for CRT are, by the nature of their disease process, prone to VA and CRT may not always be responsible for an increase in arrhythmia burden.

### Differences in CRT and ICD populations

Patients with devices implanted for secondary prevention, ion channelopathy, HCM, and congenital heart disease were excluded to facilitate comparison between the two etiologies of heart failure most commonly leading to device implantation. Overall, the two groups were reasonably well matched. However, there were differences between these groups, which requires further discussion. NYHA class was significantly higher in the CRT-D group. This is not unexpected given the criteria for CRT implantation in the UK stipulates NYHA class III-IV symptoms (ref NICE guidelines) (Appraisal Committee members and NICE project team, 2007), whereas heart failure symptoms need not be severe for ICD implantation. As a consequence, one might expect more severe underlying heart dysfunction in the CRT cohort, putting them more at risk of arrhythmia, but this is not borne out by the echocardiographic data from these two groups in the present study (including LV systolic function), which showed no significant difference in the mean EF, including the sub-group analysis. We therefore speculate this symptomatic difference between the groups is unlikely to influence the results of the study.

Patients in the ICD arm had a higher proportion of males and patients with an ischemic substrate. The two cohorts in the analysis were well matched in respect to renal function. This is important as renal disease is a well-recognized independent risk factor for sudden death in heart failure patients (Korantzopoulos et al., 2009; Kreuz et al., 2010; Shamseddin and Parfrey, 2011) and often co-exists in patients with impaired LV function. Electrolyte abnormalities as a result of renal disease may also predispose to VA (Whitman et al., 2012) and therefore act as a confounding factor. The Cox regression analysis in our study identified presence of CKD as one of the independent predictors of VT/VF. Advanced age and male-sex were the other independent predictors of sustained VAs. Additional comorbidities, including presence of coronary disease and CKD with advancing age might explain, age being independent predictor of sustained VA.

## Limitations

This study is limited by its retrospective nature of data collection. Some baseline characteristics (gender and drugs) were not well matched between the two groups which may have acted as confounders. Moreover the sizes of the cohorts studied were small and this is well acknowledged as one of the limitations of this study. The use of device therapies (ATP or shocks) as a surrogate for sustained VA is likely to be highly sensitive, but not specific. Nominal settings for detection of VF on most devices is relatively short (for example 18 out 24 intervals on Medtronic devices or minimum 1 s of tachycardia on Boston Scientific devices) meaning that therapy is delivered very early after the onset of tachycardia, which may have terminated spontaneously shortly afterwards. Thus, using this measure is likely to overestimate the real proportion of sustained arrhythmia, but one would expect this to be similar between the two groups as long as programmed device detection criteria was also similar.

## Author contributions

All authors contributed to manuscript concept/design, data analysis/interpretation, drafting article, critical revision of article, approval of article, statistics, and data collection.

### Conflict of interest statement

The authors declare that the research was conducted in the absence of any commercial or financial relationships that could be construed as a potential conflict of interest.
